# Comparison of *K*-Means and Fuzzy *c*-Means Algorithm Performance for Automated Determination of the Arterial Input Function

**DOI:** 10.1371/journal.pone.0085884

**Published:** 2014-02-04

**Authors:** Jiandong Yin, Hongzan Sun, Jiawen Yang, Qiyong Guo

**Affiliations:** 1 Sino-dutch Biomedical and Information Engineering School of Northeastern University, Shenyang, Liaoning, China; 2 Department of Radiology, Shengjing Hospital of China Medical University, Shenyang, Liaoning, China; Vanderbilt University, United States of America

## Abstract

The arterial input function (AIF) plays a crucial role in the quantification of cerebral perfusion parameters. The traditional method for AIF detection is based on manual operation, which is time-consuming and subjective. Two automatic methods have been reported that are based on two frequently used clustering algorithms: fuzzy *c*-means (FCM) and *K*-means. However, it is still not clear which is better for AIF detection. Hence, we compared the performance of these two clustering methods using both simulated and clinical data. The results demonstrate that *K*-means analysis can yield more accurate and robust AIF results, although it takes longer to execute than the FCM method. We consider that this longer execution time is trivial relative to the total time required for image manipulation in a PACS setting, and is acceptable if an ideal AIF is obtained. Therefore, the *K*-means method is preferable to FCM in AIF detection.

## Introduction

Perfusion magnetic resonance imaging (MRI) can be used to assess cerebral hemodynamic parameters for non-invasive diagnosis and staging of disease and for treatment monitoring. This method involves monitoring of rapid changes in signal intensity over time for a tracer passing though the capillary bed. Quantitative analysis using dynamic susceptibility contrast (DSC) MRI perfusion requires determination of the arterial input function (AIF), which is the concentration of the contrast agent over time in a brain-feeding artery [1], [2]. It is used in the deconvolution of tissue time–concentration curves to obtain hemodynamic maps of cerebral blood flow (CBF), cerebral blood volume (CBV), and mean transit time (MTT) [3]–[10]. Thus, AIF profile has a profound effect on final calculation of cerebral blood parameters. Previous methods used to obtain AIF in clinical practice require manual selection, which is subjective and time-consuming [11]–[14], and operators must be specially trained. Then, two automatic methods were developed by Mouridsen et al. [7] and Murase et al. [15] based on two widely used multivariate statistical techniques: fuzzy *c*-means (FCM) and *K*-means clustering. Although several comparative analyses of FCM and *K*-means clustering algorithms have been reported [16]–[18], to the best of our knowledge, no similar study has been carried out on cerebral perfusion field and it is still unclear which method can provide better AIF for subsequent hemodynamic quantification. Hence, in the present study we compared the accuracy, duration, and reproducibility of FCM and *K*-means cluster analysis for AIF detection using both simulated and clinical data. Our ultimate aim was to obtain improvements in perfusion quantification.

## Methods

### 1. Simulation Data

All the experiments are carried out on an off-line personal computer (Inter Core i3 M350 CPU processor, 2.27 GHz operating frequency, 2.0 GB RAM memory capacity, Microsoft Windows 7 operating system). A MATLAB program was developed in our department for comparison between FCM and *K-*means clustering using both simulated data and clinical data.

The simulation was set up as reported by Peruzzo et al. [5]. The simulation data contained:

Six “true” arterial voxels not affected by partial volume effects (PVEs);
16 “false” arterial voxels;
440 voxels simulating normal gray matter (GM) tissue;
440 voxels simulating pathological GM tissue;
600 voxels simulating normal white matter (WM) tissue; and
400 voxels contaminated by PVEs.

The true AIF comprises the main peak (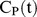) and subsequent recirculation (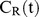):
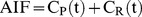
(1)

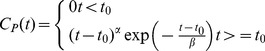
(2)

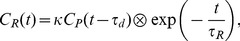
(3)where 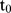 is the arrival time of contrast agent, 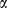 is a measure of inflow velocity steepness, 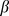 is the washout velocity, the symbol “
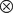
” represents the convolution operation, 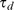 is the delay between the principal peak and recirculation, 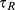 is the time constant for the function accounting for recirculation dispersion, and 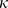 is a constant that ensures that the recirculation peak is the third part of the main peak. We used values of 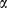 = 3.0, 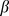 = 1.5 s, 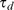 = 8 s, 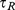 = 30 s [5], [15], [19], [20], and 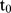 = 26 s, which closely approximates the contrast agent arrival time for our clinical perfusion data.

The residue function R(t) was modeled using a gamma variate function to simulate the presence of bolus dispersion.
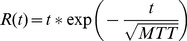
(4)where MTT equals the ratio of CBV to CBF.

Then the relationship between contrast concentration 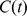 and signal intensity 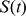 was established using the following equations:
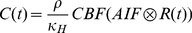
(5)

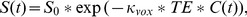
(6)where 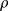 = 1.04 g/ml is the density of brain tissue, 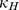 = 0.73 is a correction factor that takes into account the difference in hematocrit between large vessels used for AIF determination and small vessels such as capillaries and arterioles, 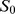 is the image baseline intensity, which equals 100 here [19], and 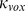 is selected to result in a 40% signal peak decrease from baseline for normal GM, which corresponds to values typically found in clinical cases. The scan time for simulation experiments was 90 s with an echo time (TE) of 0.03 s to match the scan parameters for our clinical perfusion data.

Different tissue types and pathological states were simulated using the following parameters: normal GM tissue, CBV = 4 ml/100g, MTT = 4±0.33 s; normal WM tissue, CBV = 2 ml/100 g, MTT = 5.45±0.33 s; and pathological GM tissue, CBV = 3.3 ml/100 g, MTT = 10±0.7 s. Sixteen pseudo-AIFs were generated by changing 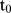 from 27 to 30 s and 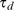 from 9 to 12 s in increments of 1.0 s. Voxels affected by PVE were generated by linear combinations of a true arterial signal and signal for one of the different tissues, with weights selected at random.

### 2. Clinical Data

#### 2.1 Ethics statement

Ethical clearance was obtained for this study from the Ethics Committee of Shengjing Hospital of China Medical University (No. 2013PS113K) and written informed consents were obtained from all participants prior to the study onset.

#### 2. 2 Acquisition protocol

Forty-two volunteers (27 males and 15 females) aged 23–69 years (average = 49.5) agreed to participate in this study and underwent DSC MR imaging. Before the scans, all of the participants were told to abstain from caffeine drinking and eating to minimize their physiologic fluctuations. All of the subjects were confirmed healthy by a senor neurologist.

The imaging data were acquired using a 3.0 T whole-body MR scanner with multichannel capabilities (MAGNETOM Verio; Siemens Medical Solution, Erlangen, Germany). Before the contrast material was injected, the diffusion-weighted images (DWI), conventional T1- and T2-weighted images (T1WI and T2WI), and T1-weighted contrast material-enhanced images were acquired with the morphological scanning sequences. For the DSC perfusion imaging, a single-shot echo planar imaging (EPI) sequence was performed with the following parameters: repetition time (TR) = 1500 ms, echo time (TE) = 30 ms, matrix = 128×128, field of view (FOV) = 23×23 cm, slice thickness = 4 mm, spacing between slices = 5.2 mm, slice number = 19, acquisition type = 2D, number of phase encoding steps = 127, transmitting coil = body, and flip angle = 90°. Sixty-two whole-head images were obtained (scanning time = 93 s) per subject and one slice covered the horizontal part of the right middle cerebral artery (MCA). At the seventh time point, a gadolinium-based contrast agent (Gadovist; Bayer Schering Pharma AG, Berlin, Germany) was administered at 0.1–0.2 mmol/ml per kilogram of body weight using a power injector at a rate of 4 ml/s, followed by an equal volume of saline flush at the same injection speed.

The magnetization state was not steady at the beginning of perfusion scanning, so the first two images were discarded and the time 0 was assigned to the third acquired volume. Therefore, 60 volume images were used in the subsequent quantitative analysis.

### 3. AIF Determination

#### 3.1 Clustering analysis on simulation data

First, the signal intensity was converted to contrast agent concentration using the inversion formula of Eq. (6) [7], [8]. Then FCM and *K*-Means clustering algorithms were applied to the converted data respectively according to the mathematical principles outlined by Murase et al. [15] and Mouridsen K et al. [7]. These clustering techniques are very mature, so it is not described in detail here. As done by Mouridsen et al. [7] and Peruzzo D et al. [5], the number of clusters was set to 5. For the mean curve of each cluster, the peak value (PV), the time to peak (TTP), and the full-width half-maximum (FWHM) were computed, then a measure *M* = PV/(TTP*FWHM) was calculated. The cluster with the highest *M* value was deemed as the most appropriate one for determining AIF from the mean time–concentration curve [15].

#### 3.2 Clustering analysis on clinical data

First, physiological fluctuations (breathing, heartbeats, etc.) and involuntary motions by the subject were difficult to avoid during the acquisition of 60 bolus tracking volumes, which resulted in misalignments of the volume images at different time points. The images were realigned to the first pre-contrast volume using SPM (http://www.fil.ion.ucl.ac.uk/spm/) (version, SPM99) and INRIAlign 1.01 [21]–[23]. No smoothing was performed on any of the images.

Second, the slice contained the right horizontal MCA was extracted from the first volume image. The same principle was applied to the given slice for calculating contrast agent concentration. The arterial regions had small areas, so only a small fraction of the entire set of time-concentration curves corresponded to artery pixels [13]. Most pixels represented tissue voxels and exhibited small changes in their signal intensity. It was necessary to eliminate these weak pixels to optimize AIF detection [5], [7]. Thus, the area under the time-concentration curve was computed for each pixel and the P_AUC-L_ percentage of the curves with the smallest areas were excluded [5], [7].

Third, during the scanning of perfusion images, some fluctuating curves were obtained because of shifts in voxels, PVEs, physiological pulsations, and other effects. These irregular curves would produce poor estimates of the true AIF [7]. Thus, the following standard roughness measurement method was used and the P_rough_ percentage of the remaining curves with the largest integral values were excluded [7].
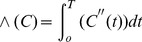
(7)


As reported by Mouridsen K et al. [7], the value of P_AUC-L_ and P_rough_ was assigned to 0.90 and 0.25.

Finally, the FCM and *K*-means were applied to the residual curves. As done previously, the number of clusters was still set to 5, and the AIF cluster was determined using the measure *M* = PV/(TTP*FWHM) again.

### 4 Statistical Analysis

#### 4.1 Simulation data

Several parameters were computed to evaluate the performance of the two methods for AIF detection.

First, PVE was calculated as the percentage non-arterial signal in voxels selected as the AIF cluster by the algorithms. Low PVE indicates that arterial voxels can be well discriminated by the corresponding algorithm. In addition, FWHM, TTP, and PV were compared for the FCM and *K*-means clustering methods to evaluate the AIF detection accuracy [3]. An estimated AIF with lower PVE exhibits greater PV and smaller FWHM, and an earlier TTP indicates a shorter bolus delay [3].

Second, integrated AIF curves correlate well with the relative CBV (rCBV) [2], [5], so the area under the AIF curve (AUC) is another important parameter in assessing estimated AIFs.

Finally, the difference between estimated and true AIFs was computed as the root mean square error (RMSE).
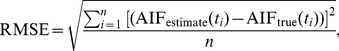
(8)where *n* is the scan time (90 s).

#### 4.2 Clinical data analysis

Similar to the simulation data, the influence of the two algorithms on AIF detection accuracy was evaluated by comparing the shape parameters (TTP, PV, FWHM) and AUC.

Second, the measurement reproducibility of each algorithm was validated by comparing AIF results calculated independently 50 times in succession. Robustness, defined as the variance for AIF curves, was quantitatively assessed according to
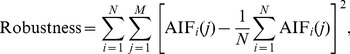
(9)where *M* is the number of time points (60), and *N* is the number of calculation repeats (50) [13].

Third, the computational times for AIF detection were compared between FCM and *K-*means clustering methods.

The comparisons were performed using a paired-samples *t*-test. A *P*-value <0.05 was considered significantly different. The statistical analysis was conducted using SPSS (SigmaStat, 2.03, Inc., Chicago, IL).

## Results

### 1. Simulated Data

**Figure 1:**
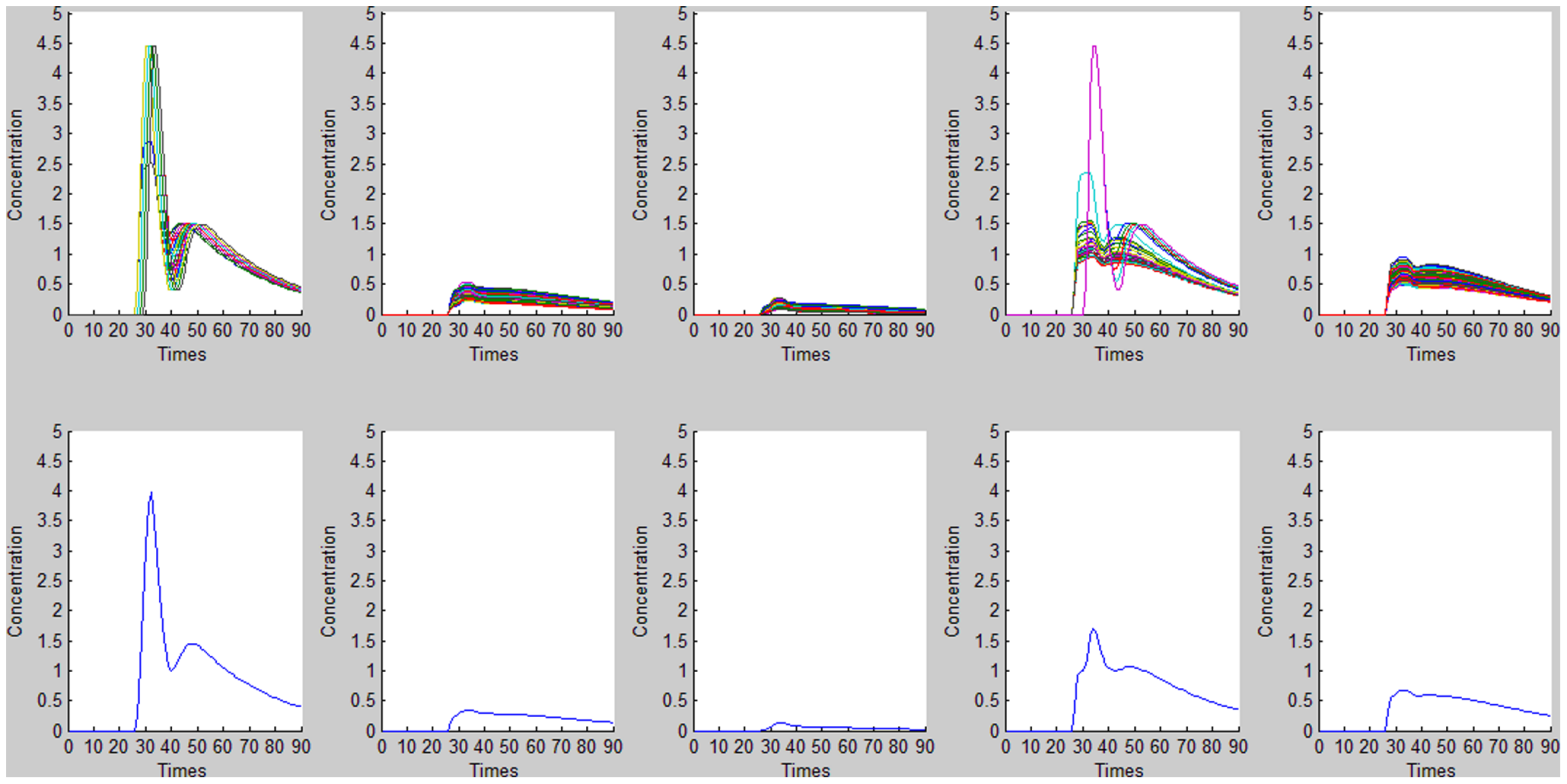
FCM cluster analysis results for AIF detection. Top: time–concentration curves for different clusters. Bottom: mean curve for *M* values of 0.0182, 1.9697e-004, 1.7418e-004, 0.0016, 3.9782e-004, respectively. The mean curve for *M* = 0.0182 was thus selected to represent the estimated AIF.

**Figure 2:**
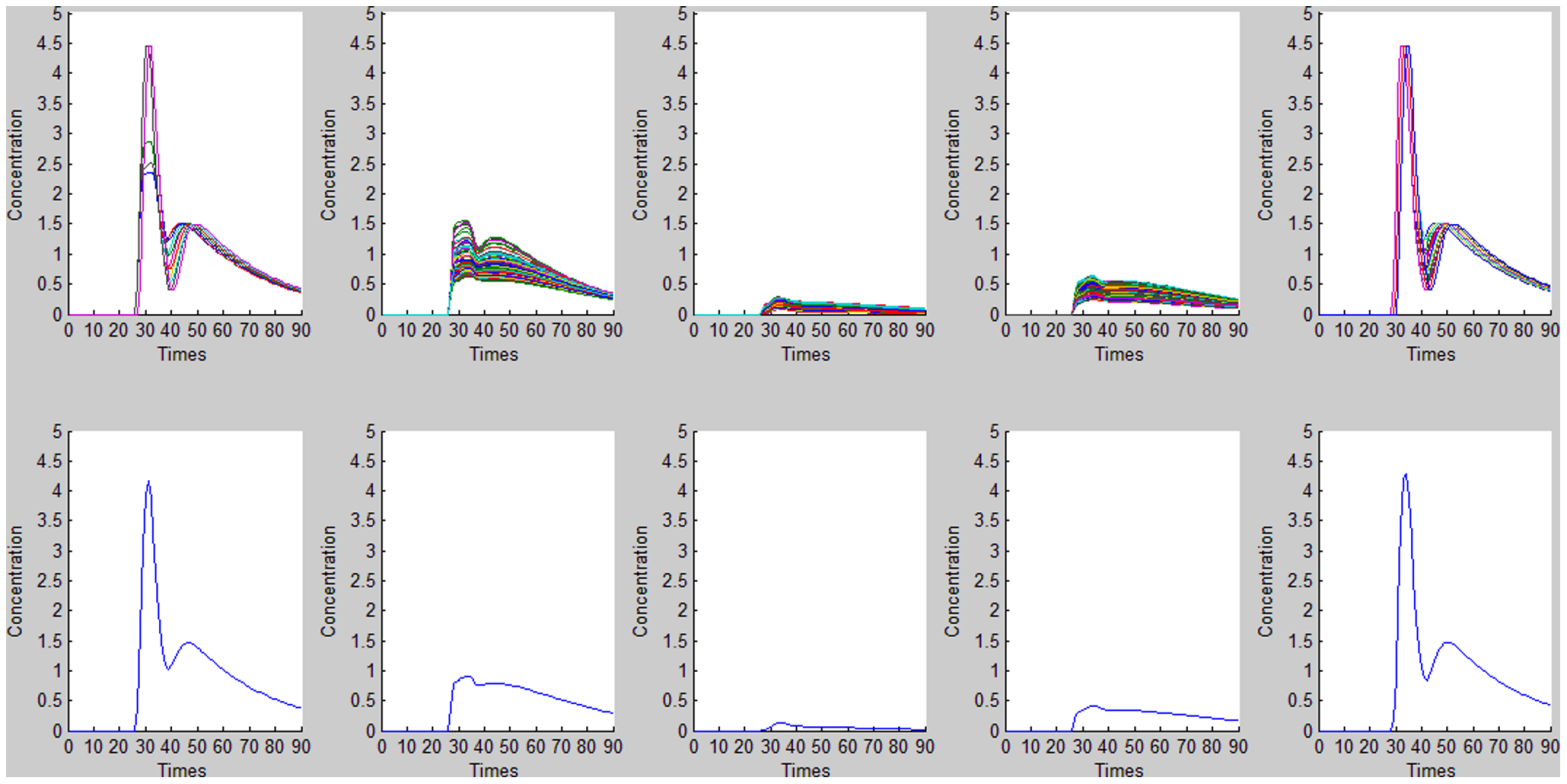
K-means cluster analysis results for AIF detection. Top: time–concentration curves for different clusters. Bottom: mean curve for *M* values of 0.0212, 5.8216e-004, 1.7180e-004, 2.3354e-004, 0.0205, respectively. The mean curve for *M* = 0.0212 was thus selected to represent the estimated AIF.

**Figure 3:**
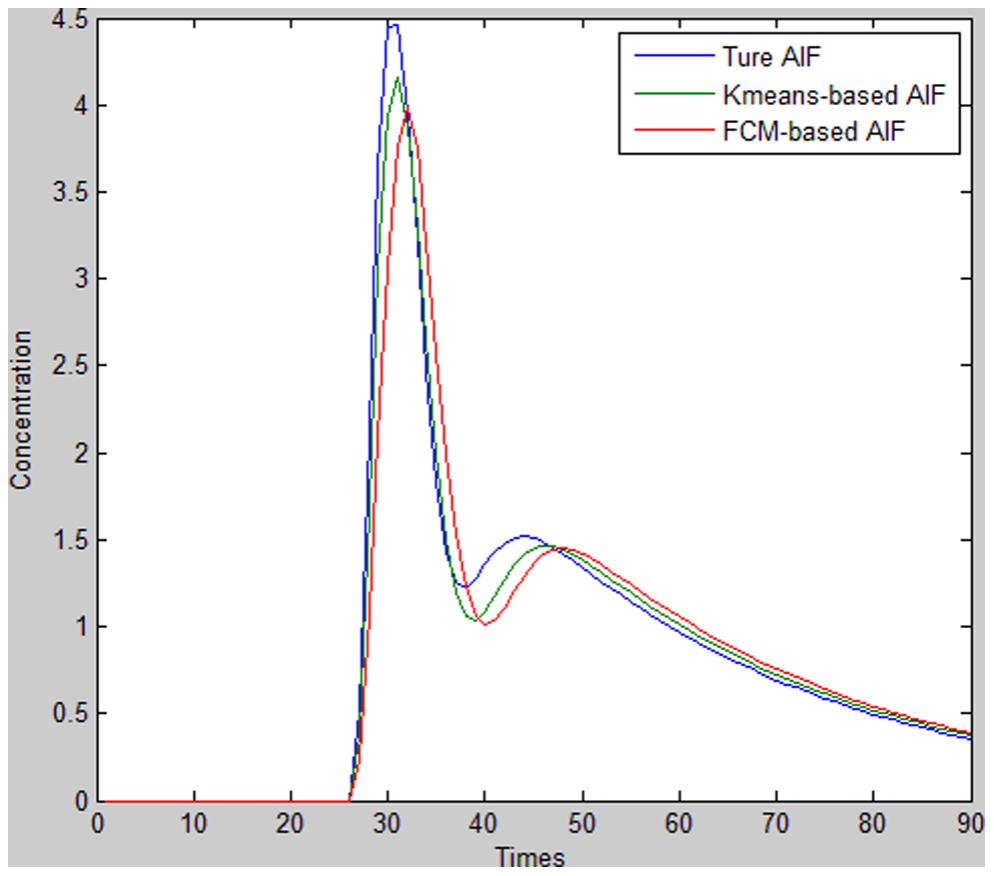
Comparison of the true AIF, K-means-based AIF, and FCM-based AIF.

Results for the two algorithms for AIF detection are shown in Figure 1–3 and Table 1.

**Table 1:** Comparison of AIFs based on the two clustering methods and the true AIF.

AIF	PVE	PV	TTP	FWHM	AUC	RMSE	M
**FCM-based**	0.7857	3.9895	30.95	7.0710	75.1458	0.3295	0.0182
***K*** **-Means-based**	0.6842	4.1607	29.88	6.5771	75.4032	0.2028	0.0212
**True**	0	4.4592	29.51	6.2016	76.8669	0	0.0244

The results reveal that the *K*-means method is less affected by PVE, as confirmed by the lower PVE value. This method also exhibits larger AUC, higher PV, and narrower FWHM compared to the FCM method. In addition, TTP is earlier for *K*-means clustering than for FCM, which means that the *K*-means method is less influenced by tracer transport delays. The lower RMSE and visual inspection of Figure 3 indicate that the *K*-means-based AIF curve is closer to the true AIF curve than FCM-based AIF is. The *M* value for the true AIF is also closer to the *K*-means than the FCM value, confirming that the AIF derived from *K*-means clustering is more accurate than the FCM-derived AIF.

### 2. Clinical Data

**Figure 4:**
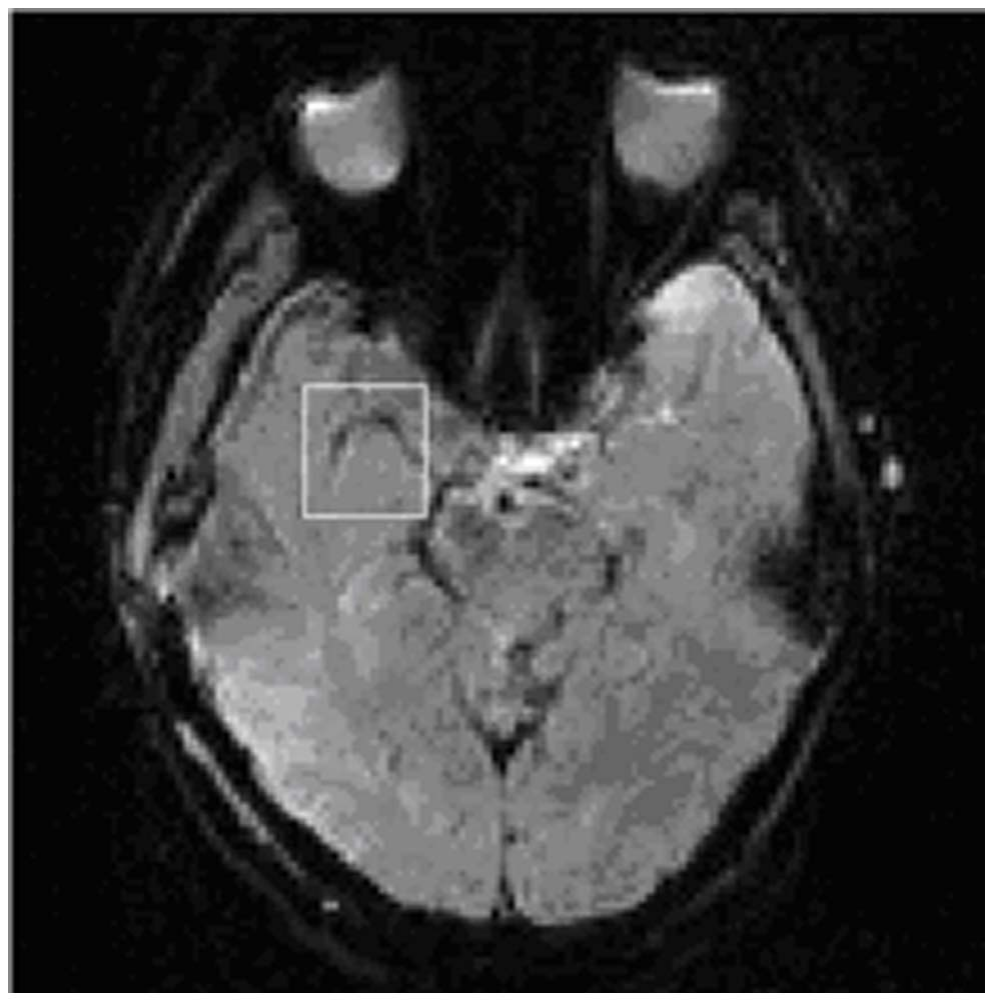
Image of a slice containing the right MCA (white rectangle) extracted from the first dynamic of perfusion scans for clustering analysis.

**Figure 5:**
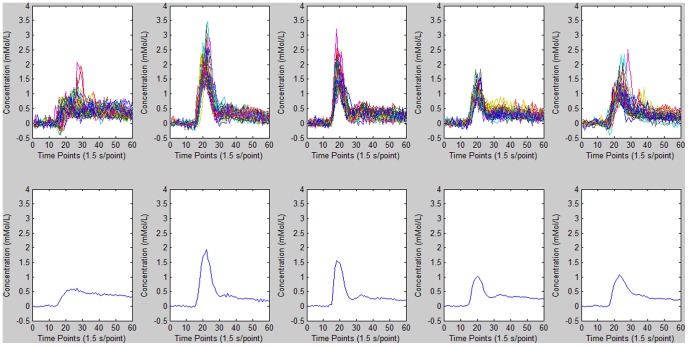
AIF detection results for FCM cluster analysis. Top: time–concentration curves for different clusters. Bottom: mean curves for *M* values of 5.7745e-004, 0.0145, 0.0135, 0.0070, and 0.0047, respectively. The mean curve for *M* = 0.0145 was thus selected to represent the estimated AIF.

**Figure 6:**
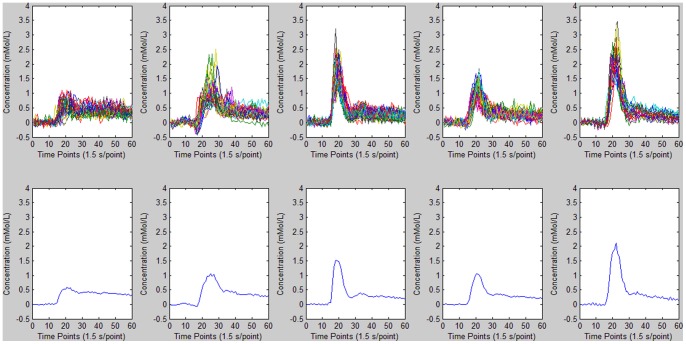
*K*-Means cluster analysis results for AIF detection. Top: time–concentration curves for different clusters. Bottom: mean curve for *M* values of 6.7346e-004, 0.0036, 0.0138, 0.0066, and 0.0148, respectively. The mean curve for *M* = 0.0148 was thus selected to represent the estimated AIF.

**Figure 7:**
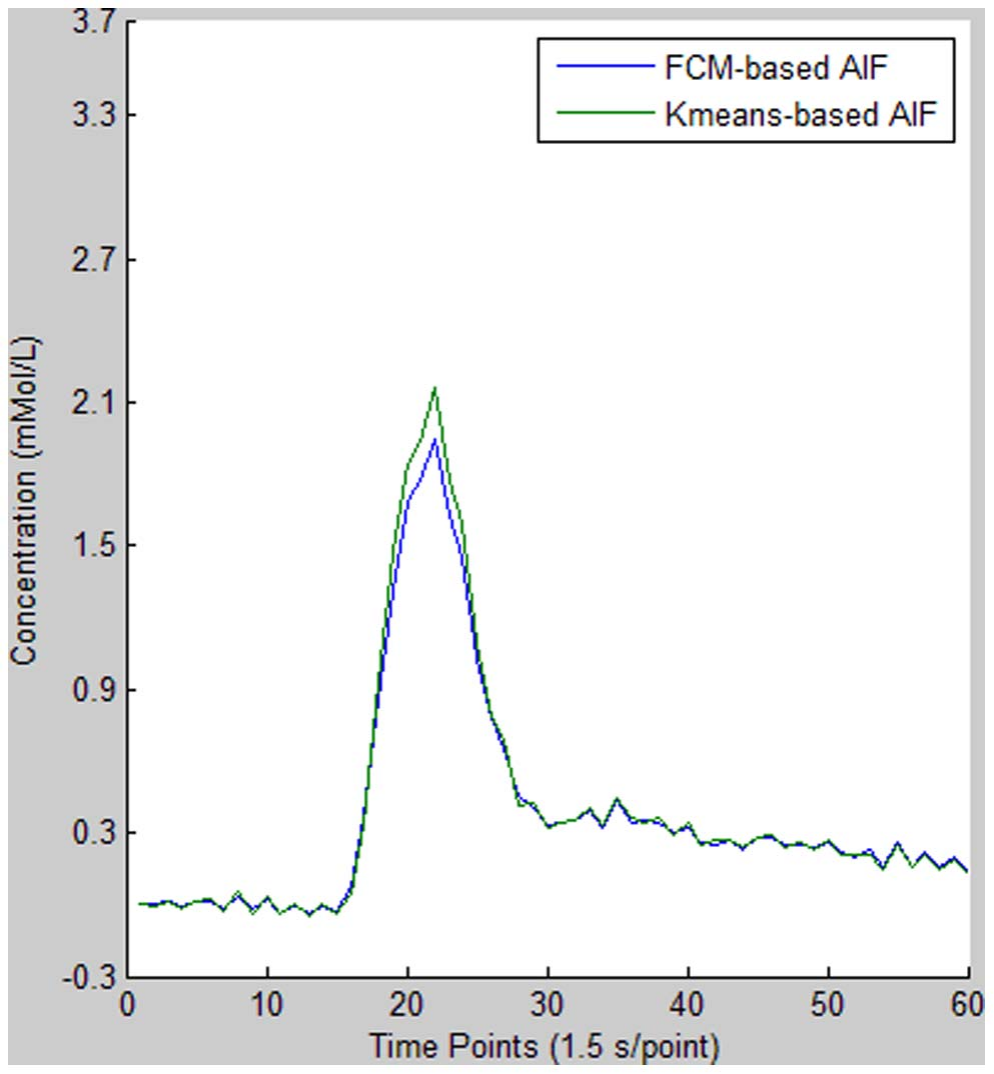
Comparison of AIFs derived from the FCM and *K*-means clustering methods. Relative to FCM, the *K*-means-based AIF shows similar TTP, higher PV, larger AUC, and narrower FWHM.

**Figure 8:**
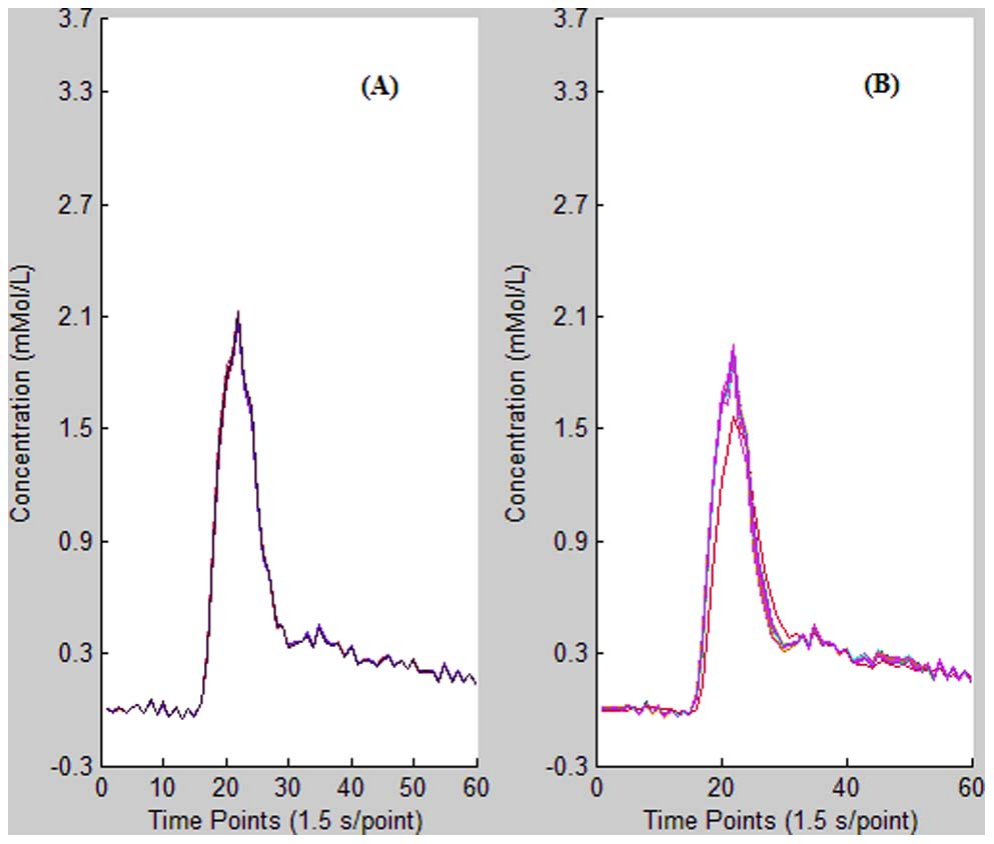
Comparison of AIF detection reproducibility for (A) *K*-means and (B) FCM clustering methods. Both algorithms were independently executed 50 times in succession. The robustness is 0.131 for the *K*-means method and 10.901 for FCM. Hence, *K*-means clustering shows better reproducibility than FCM for AIF detection.

According to the predetermined steps, AIFs were obtained for each participant using the *K*-means and FCM methods. We use a 37-year old man as an example to describe differences between the AIF detection methods. Figure 4 shows an optimal slice for clustering analysis. Figures 5 and 6 show *K*-means and FCM clustering results, respectively. Figure 7 compares AIFs derived from the two algorithms. Figure 8 shows the reproducibility of the different clustering methods for AIF detection.

Statistical results are presented in Table 2. AIFs measured using the *K*-means algorithm have higher PV, in agreement with the simulation result. The difference is significant (*P*<0.05). TTP values calculated using the FCM and *K*-means methods are in good agreement and the difference is not significant (*P*>0.05). The mean FWHM is slightly narrower for *K*-means-based AIF than for FCM-based AIF, but the difference is not significant (*P*>0.05). Both mean AUC and *M* value are significantly higher for the *K*-means than for the FCM method (*P*<0.05). The variance (robustness) for AIF curves is significantly lower for the *K*-means method compared to FCM (*P*<0.05), indicating that the calculation–recalculation reproducibility is better for *K*-means cluster than for the FCM method for AIF detection. The mean execution time is significantly longer for the *K*-means cluster method than for FCM cluster analysis (*P*<0.05).

**Table 2:** Statistical analysis of the results derived from FCM and *K*-means methods for AIF detection.

Method	Shape parameters			AUC (Mean± SD)	M value (Mean±SD)	Robustness (Mean±SD)	Computational time (Mean±SD)
	TTP (Mean±SD)	PV (Mean± SD)	FWHM (Mean± SD)				
***K*** **-Means**	20.935±0.321	2.171±0.189	6.76±0.37	23.76±1.80	0.0155±0.0040	1.391±0.712	0.2431±0.0928
**FCM**	20.982±0.943	1.950±0.462	6.92±0.92	19.66±4.01	0.0134±0.0047	7.937±2.764	0.1407±0.0637
**Statistical analysis**	*P*>0.05	*P*<0.05	*P*>0.05	*P*<0.05	*P*<0.05	*P*<0.05	*P*<0.05

## Discussion

AIF must be determined in advance to quantify the perfusion parameters such as CBV, CBF, and MTT. Conventional methods require that a ROI is drawn on the MCA or internal carotid artery (ICA) manually, before calculating the AIF by averaging the time-concentration curves of all the pixels in the ROI. However, this manual method had some limitations during AIF determination. First, the calculation results are subjective, so there is a lack of consistency among different operators and among different time points with the same operator. Second, this method is time-consuming and unfeasible in some cases, such as acute stroke, where the confirmation of the location and the amount for salvageable tissue can be used to identify cases that may benefit from thrombolytic therapy, but the treatment must be initiated rapidly [24]–[26], so the immediate acquisition of perfusion maps is essential. Thus, an objective and rapid method for AIF detection would be of great importance.

Several automatic methods have been developed to solve the problems associated with manual operation. Early automatic methods were based on the characteristic shapes of contrast agents in the artery [9], [15] and were easy to understand and implement. However, they only used shape parameters of time-course curves, so there was a risk that a suboptimal AIF might be selected. Thus, extra information was obtained from DSC images and used to identify AIF based on various multivariate statistical methods, such as principal components analysis and cluster analysis. Cluster analysis was used to partition bundles of time–concentration curves into several groups. Curves from the same group had common shape characteristics, which could be distinguished from those of other groups. Cluster analysis included several different algorithms. Two frequently used algorithms, FCM and *K*-means clustering, have been used for automatic AIF determination independently [7], [15]. However, to the best of our knowledge, there has been no comparative analysis of *K*-means and FCM clustering techniques for AIF detection and it is not clear which performs better.

Therefore, we compared the performance of FCM and *K*-means cluster analysis for AIF detection using both simulation and clinical data. The results demonstrate that AIFs obtained using *K*-means clustering have a higher peak than AIFs obtained using FCM analysis, and the difference was significant for clinical data. Thus, *K*-means-based AIF determination might be less affected by mixing of the arterial signal with signals from surrounding tissue [3], [27], so the resulting AIF approaches optimality. AUC was higher for the *K*-means method than for FCM and was closer to the true AIF for simulation data; the *K*-means–FCM difference was significant for clinical data. This indicates that AIF determination based on the *K*-means method is affected by minimal partial volume averaging [3]. The higher peak and larger integrated bolus curve for the *K*-means-based AIF indicate that this method yields the measurements more close to true AIFs [3], so it should facilitate more accurate quantitative determination of CBF, CBV, and MTT. To compare the reproducibility, each algorithm was executed 50 times for the same batch of data. The results reveal better reproducibility for *K*-means clustering than for FCM analysis. It is known that erratic AIFs lead to non-reproducible quantification of cerebral parameters, which undermines the diagnosis and tracking of disease. Thus, compared to FCM clustering, the *K*-means method is preferable for AIF determination.

Finally, the question of the computational time requirements of each method needs to be addressed. The results demonstrated that the mean execution time was relatively longer with the *K*-means method and the difference was significant. This appeared to be a drawback of *K*-means clustering, but this was not the case. In current PACS environments, the total execution time required for radiodiagnosis includes the duration of image downloading from the PACS server, image post-processing on a local workstation, and image unloading to the PACS server. The entire operation process takes a few minutes to complete, possibly even more than ten minutes. Relative to the total duration of image manipulation in PACS settings, the extra time required to execute the *K*-means method compared with the FCM method appears negligible. Thus, the extra execution time did not limit the use of the *K*-means method for AIF determination in clinical practice.

It must be emphasized that there were three limitations in this research. First, one limitation was the number of subjects who participated in perfusion imaging because only 42 subjects were included in the statistical analysis. This limited number of cases might result in statistical uncertainty [28]. Therefore, it is necessary to increase the number of subjects in similar studies in the future. Second, all of the participants involved in this study were healthy and subjects with abnormalities were not included. Thus, the clinical efficacy was not validated for patients with neurological diseases, which means that it is necessary to further assess the feasibility and efficiency of this method by adding DSC images of abnormal cases with acute stroke, artery stenosis, and other abnormalities. Finally, we only evaluated the two most widely used clustering algorithms, so it is still unclear whether there are significant differences among other clustering algorithms used for AIF detection. Thus, it is necessary to compare more different types of clustering algorithms to identify the most suitable clustering method for AIF determination.

In conclusion, the *K*-means method yields more accurate and reproducible AIF results compared to FCM cluster analysis. The execution time is longer for the *K*-means method than for FCM, but acceptable because it leads to more robust and accurate follow-up hemodynamic maps.
